# Serum Angiopoietin-1 and -2 Levels Discriminate Cerebral Malaria from Uncomplicated Malaria and Predict Clinical Outcome in African Children

**DOI:** 10.1371/journal.pone.0004912

**Published:** 2009-03-20

**Authors:** Fiona E. Lovegrove, Noppadon Tangpukdee, Robert O. Opoka, Erin I. Lafferty, Nimerta Rajwans, Michael Hawkes, Srivicha Krudsood, Sornchai Looareesuwan, Chandy C. John, W. Conrad Liles, Kevin C. Kain

**Affiliations:** 1 McLaughlin-Rotman Centre for Global Health, McLaughlin Centre for Molecular Medicine, University Health Network, University of Toronto, Toronto, Ontario, Canada; 2 Faculty of Tropical Medicine, Mahidol University, Bangkok, Thailand; 3 Department of Paediatrics and Child Health, Makerere University, Kampala, Uganda; 4 Department of Pediatrics, University of Minnesota Medical School, Minneapolis, Minnesota, United States of America; 5 Division of Infectious Diseases, Department of Medicine, University of Toronto, Toronto, Ontario, Canada; BMSI-A*STAR, Singapore

## Abstract

**Background:**

Limited tools exist to identify which individuals infected with *Plasmodium falciparum* are at risk of developing serious complications such as cerebral malaria (CM). The objective of this study was to assess serum biomarkers that differentiate between CM and non-CM, with the long-term goal of developing a clinically informative prognostic test for severe malaria.

**Methodology/Principal Findings:**

Based on the hypothesis that endothelial activation and blood-brain-barrier dysfunction contribute to CM pathogenesis, we examined the endothelial regulators, angiopoietin-1 (ANG-1) and angiopoietin-2 (ANG-2), in serum samples from *P. falciparum*-infected patients with uncomplicated malaria (UM) or CM, from two diverse populations – Thai adults and Ugandan children. Angiopoietin levels were compared to tumour necrosis factor (TNF). In both populations, ANG-1 levels were significantly decreased and ANG-2 levels were significantly increased in CM versus UM and healthy controls (p<0.001). TNF was significantly elevated in CM in the Thai adult population (p<0.001), but did not discriminate well between CM and UM in African children. Receiver operating characteristic curve analysis showed that ANG-1 and the ratio of ANG-2∶ANG-1 accurately discriminated CM patients from UM in both populations. Applied as a diagnostic test, ANG-1 had a sensitivity and specificity of 100% for distinguishing CM from UM in Thai adults and 70% and 75%, respectively, for Ugandan children. Across both populations the likelihood ratio of CM given a positive test (ANG-1<15 ng/mL) was 4.1 (2.7–6.5) and the likelihood ratio of CM given a negative test was 0.29 (0.20–0.42). Moreover, low ANG-1 levels at presentation predicted subsequent mortality in children with CM (p = 0.027).

**Conclusions/Significance:**

ANG-1 and the ANG-2/1 ratio are promising clinically informative biomarkers for CM. Additional studies should address their utility as prognostic biomarkers and potential therapeutic targets in severe malaria.

## Introduction

Although greater than 500 million *Plasmodium falciparum* malaria infections are estimated to occur each year, only a small proportion of patients progress to severe and potentially fatal complications such as cerebral malaria (CM) [1], [2], [3], [4]. However, the mechanisms underlying CM are poorly understood, and limited prognostic tools are available to determine which infected individuals will progress to cerebral complications [5], [6], [7], [8].

The discovery of a reliable laboratory test that accurately identifies individuals with, or at risk of, CM would be valuable. The capacity for early detection and intervention in cases of severe malaria and CM would have clinical and economic impact, particularly in resource-poor settings where effective allocation of limited health resources is essential. Several studies have examined the correlation of serum markers, such as cytokines, with severe and complicated malaria. Elevated levels of TNF have been associated with severe malaria [9], [10], [11], [12], [13], [14] and were identified as a predictor of mortality in CM [10], [11]. However, other studies have challenged these findings and reported that TNF levels do not correlate with disease severity [15], [16].

Endothelial cell activation and dysfunction have been implicated in the pathogenesis of CM, in which the endothelium responds to parasite-induced inflammation and mediates parasitized erythrocyte sequestration, especially in vital organs such as the brain [17]. Endothelial activation markers, such as endothelial microparticles, vonWillebrand factor and soluble cell-adhesion molecules (sCAMs), including soluble intercellular adhesion molecule-1 (sICAM-1), soluble vascular cell adhesion molecule-1 and soluble endothelial leukocyte adhesion molecule-1, are increased in malaria and have been positively correlated with disease severity [14], [16], [18], [19], [20], [21]. However the role of sCAMs in the pathophysiology of malaria is unclear, and circulating levels of sCAMs may not accurately reflect expression in vascular beds [16]. Furthermore, it is unclear whether these markers are useful in predicting disease progression or outcome [16], [18], [19], [20].

In addition to systemic endothelial activation, recent work has focused on mechanisms by which malaria may compromise the structural and functional integrity of the blood-brain-barrier (BBB), leading to leakage of plasma proteins, perivascular edema and neuronal injury [22], [23], [24], [25], [26]. Angiopoietins, a recently described distinct family of angiogenic proteins, have recently been shown to play fundamental physiological roles in maintenance of vascular integrity. Angiopoietin-1 (ANG-1) is constitutively expressed and acts to maintain vascular quiescence [27]. The ANG-1 stabilizing effect is antagonized by angiopoietin-2 (ANG-2), which primes the endothelial activation response and promotes vascular permeability [27], [28]. In healthy individuals, serum ANG-1 levels are normally high, while serum ANG-2 levels are low. Consequently, an increase in ANG-2, or a dysregulation of the ANG-1/2 balance, may be associated with disease states that cause inflammation and vascular permeability [27], [28]. Specifically, elevated ANG-2 levels have been reported in patients with severe sepsis and may contribute to sepsis-related vascular leak [28], [29], [30], [31].

Based on the hypothesis that dysregulation of angiopoietins may be associated with endothelial and BBB dysfunction during malaria infection, we examined whether both ANG-1 and ANG-2 were clinically informative biomarkers for cerebral malaria. We report that angiopoietin levels were accurate biomarkers of CM and predicted mortality in African children.

## Methods

### Thai Study population

Individuals (≥13 years of age) admitted to the Hospital for Tropical Disease (Mahidol University, Thailand) for ongoing studies of anti-malarial drug efficacy were eligible for enrolment. The institutional review board of Mahidol University approved the study, and written informed consent was obtained from all patients or their legal guardians. Venous blood samples were collected from 50 patients with *P. falciparum* malaria (25 consecutive cases of UM and 25 consecutive cases of CM) prior to the initiation of standard anti-malarial therapy, and from 10 healthy controls who had negative blood smears and no history of malaria infection in the previous 6 months (Table 1). Patients with UM were defined based on a positive blood smear for *P. falciparum* without evidence for severe or complicated malaria as defined by the WHO [1]. CM was defined as *P. falciparum* infection on blood smear, unrousable coma (Glasgow coma scale ≤8) not attributable to other causes [1].

**Table 1:** Demographic information for adult malaria patients from Thailand and pediatric malaria patients from Uganda; healthy controls (HC), uncomplicated malaria patients (UM) and cerebral malaria patients (CM).

Group	Adult (Thailand)			Pediatric (Uganda)		
	N	Age	Parasites/µl	N	Age	Parasites/µl
**HC**	10	32 (25–48)	0	28	7 (3.2–12)	0
**UM**	25	22 (14–63)*	2.2×10^4^ (170-1.9×10^5^)*	67	7 (3–12)	3.3×10^4^ (48-2.4×10^5^)*
**CM**	25	25 (17–50)	3.1×10^5^ (500-2.1×10^6^)*†	69	5.4 (3.2–12) *†	4.0×10^5^ (32-9.3×10^5^)*

Age and parasitemia are presented as median (range). ^*^p<0.05 vs. HC and ^†^p<0.05 vs. UM (Kruskal-Wallis test with Dunn's multiple comparison post-test).

### Ugandan Study population

The Ugandan study population has been previously described [32]. The institutional review board at Makerere University, Faculty of Medicine (Kampala, Uganda) granted ethics approval and written informed consent was obtained from the parents or guardians of study participants. Briefly, children 4–12 years old admitted to Mulago Hospital were eligible for enrolment if they had UM or met the WHO criteria for CM: *P. falciparum* on blood smear and coma (Blantyre coma scale ≤2 or Glasgow coma scale ≤8) not attributable to hypoglycemia, convulsions, meningitis or other identifiable cause [1]. Lumbar punctures were performed to rule out meningitis/encephalitis. Children were considered to have UM if they had fever (or a history of fever within 24 hours), *P. falciparum* infection on blood smear, but no evidence of severe or complicated malaria (1). Healthy controls were recruited from the extended household areas of children with CM or uncomplicated malaria and were determined to be healthy by medical history (with no malaria history for the previous 6 months), physical examination and microscopic examination of blood smears (Table 1). Blood samples from malaria patients were drawn prior to the initiation of standard anti-malarial therapy.

Sample handling and quantification of serum biomarker levels: Serum derived from patient blood was immediately frozen, shipped on dry ice, and maintained at −80°C until use. The serum used was thawed (on ice) and re-frozen a maximum of 3 times. Serum concentrations of ANG-1, ANG-2 and TNF were measured by ELISA (R&D Systems, Minneapolis MN; TNF: eBioscience, San Diego CA). Concentrations were interpolated from 4-parameter-fit standard curves generated using a standard curve of recombinant human proteins. The upper and lower limits of detection for each assay were as follows: ANG-1 (10,000–156.25 pg/ml), ANG-2 (3,500–54.69 pg/ml) and TNF (500–7.8 pg/ml). Samples were diluted between 1∶2 to 1∶50 in assay diluent to fall within the range of the standard curves, as per the manufacturers' instructions. TNF levels in Ugandan children were measured as described [32].

### Statistical Analysis

Statistical analysis was performed using GraphPad Prism v4.03 (San Diego, CA). Serum protein levels were analyzed using a Kruskal-Wallis test, followed by Dunn's multiple comparison tests. Receiver operating characteristic (ROC) curves and area under the ROC curves were generated using (SPSS 16.0. Cutoff values were derived mathematically from the ROC curves, using the point on the ROC curve with the lowest value for the formula: (1-sensitivity)^2^+(1-specificity)^2^. Angiopoietin levels and survival outcomes were analyzed using the Wilcoxon rank-sum test. Multivariable logistic regression modeling was used to examine the independent predictive value of biomarkers on outcome (CM vs.UM) in order to account for potential confounding effects of multiple covariates (SPSS 16.0). Hosmer Lemeshow test was used to verify model goodness of fit.

## Results

### ANG-1 levels are decreased and ANG-2 levels increased in the serum of cerebral malaria patients compared to uncomplicated patients and healthy controls

**Figure 1:**
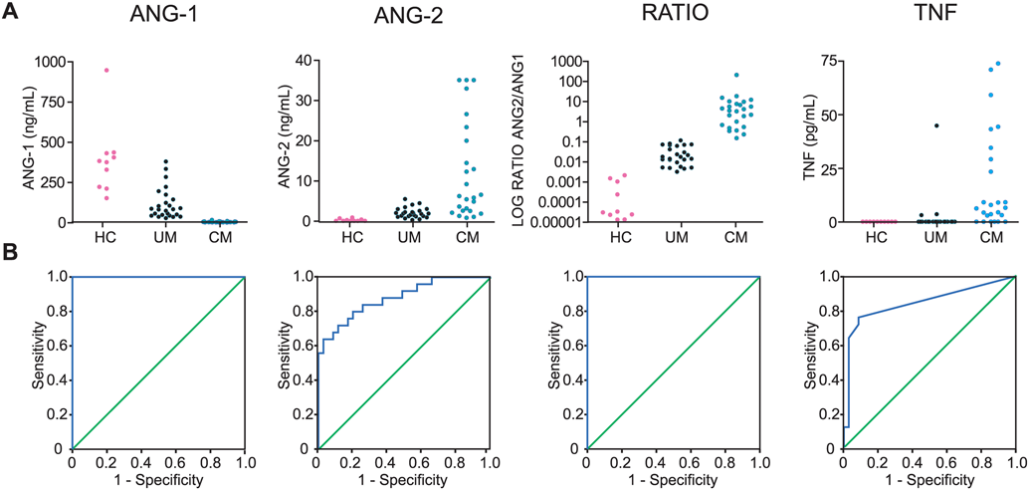
Comparison of angiopoeitin-1 and -2 levels with TNF in adult malaria patients from Thailand. A. Serum concentrations of angiopoietin-1 (ANG-1), angiopoietin-2 (ANG-2), the ratio of ANG-2∶ANG-1 (RATIO, expressed as log base 10) and tumour necrosis factor (TNF) were measured in 10 healthy controls (HC), 25 consecutive uncomplicated malaria (UM) patients, and in consecutive 25 cerebral malaria (CM) patients. B. Receiver operating characteristic curves (blue line) were generated for each test to compare CM with UM patients, with the null hypothesis (green line) that area under the curve equals 0.5.

In Thailand, serum ANG-1 levels were significantly lower in adults with CM compared to either adults with UM or healthy controls, and in adults with UM compared to healthy controls (Figure 1A; Kruskal-Wallis: p<0.001). Moreover, serum ANG-2 levels were significantly increased in adults with CM compared to adults with UM or healthy controls, as well as in adults with UM compared to healthy controls (Figure 1A; Kruskal-Wallis: P<0.001). As an additional measure, the ratio of ANG-2 to ANG-1 for each patient was found to be significantly different between healthy controls and adults with UM (Figure 1A; Kruskal-Wallis: p<0.05) and between either healthy controls or adults with UM and adults with CM (p<0.001). To compare these novel biomarkers to an established biomarker of CM, serum TNF levels were also determined. TNF was significantly increased in adults with CM compared to either adults with UM or healthy controls (Figure 1A; Kruskal-Wallis: p<0.001). However, absolute levels of TNF were very low, requiring larger sample volumes to detect.

**Figure 2:**
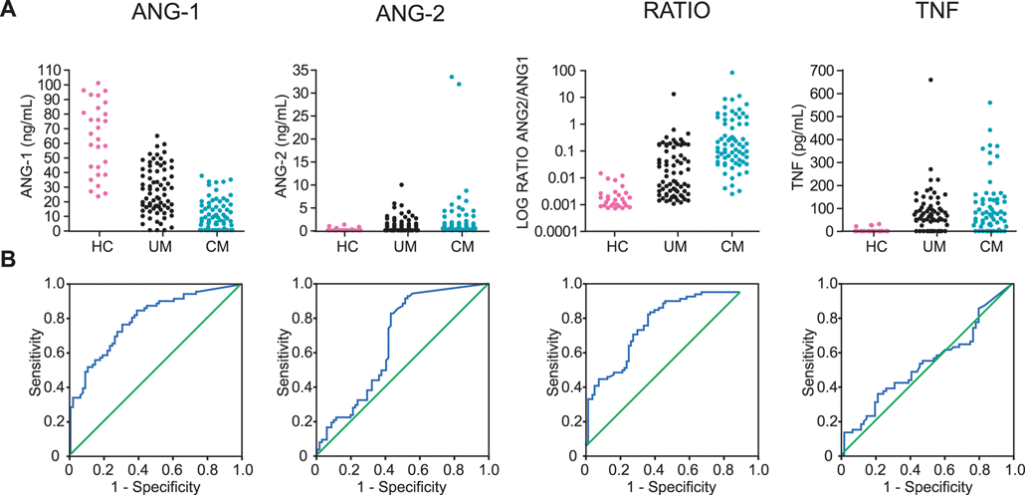
Comparison of angiopoietin-1 and -2 with TNF in pediatric malaria patients from Uganda. A. Serum concentrations of angiopoietin-1 (ANG-1), angiopoietin-2 (ANG-2), the ratio of ANG-2∶ANG-1 (RATIO, expressed as log base 10), and tumour necrosis factor (TNF) were measured in 28 healthy controls (HC), 67 uncomplicated malaria (UM) patients, and in 69 cerebral malaria (CM) patients. B. Receiver operating characteristic curves (blue line) were generated for each test to compare CM with UM patients, with the null hypothesis (green line) that area under the curve equals 0.5.

The manifestations and outcomes of severe and CM may differ between adults and children and between varying genetic backgrounds of patient and parasite populations [1], [3], [8], [16]. Therefore, the hypothesis that angiopoietin levels are informative biomarkers for CM was further examined in a larger cohort of African children. Similar to the observations in Thailand, serum ANG-1 levels were significantly decreased in Ugandan children with CM compared to Ugandan children with UM and healthy controls, and in Ugandan children with UM compared to healthy controls (Figure 2A; Kruskal-Wallis: p<0.001). Additionally, ANG-2 levels were significantly elevated in children with CM compared to children with UM and healthy controls (Figure 2A; Kruskal-Wallis: p<0.001), and between children with UM and healthy controls (p<0.01). Furthermore, as in the adult population, the ANG-2∶ANG-1 ratio was significantly higher in children with CM than in children with UM and healthy controls, and in children with UM compared with healthy controls (Figure 2A; Kruskal-Wallis: p<0.001). While TNF levels were significantly lower in healthy controls compared to children with UM and children with CM (Figure 2A; Kruskal-Wallis: p<0.001), there was no significance difference in serum TNF values between children with CM and children with UM.

Comparisons of the median and range of each serum biomarker concentration (Table 2), revealed no overlap in the ranges of ANG-1 and the ANG-2∶ANG-1 ratio measures in the CM, UM and healthy controls groups in Thai adults, indicating that these markers clearly discriminated the respective groups. However, there was some overlap in the concentration ranges in the Ugandan children with UM the Ugandan children with CM.

**Table 2:** Biomarker levels in serum of healthy controls (HC), uncomplicated malaria patients (UM) and cerebral malaria patients (CM) from adult Thai patients and pediatric Ugandan patients.

Marker	Adult (Thailand)			Pediatric (Uganda)		
	HC	UM	CM	HC	UM	CM
**ANG-1 (ng/ml)**	378 (151–946)	82.25 (27.3–379)	3.51 (0.001–15.3)	64.4 (23.5–101)	25.0 (0.39–64.9)	9.0 (0.39–37.5)
**ANG-2 (ng/ml)**	0.0089 (0.005–0.847)	1.84 (0.25–5.44)	6.19 (0.78–35)	0.068 (0.068–1.33)	0.28 (0.068–10.0)	0.83 (0.068–33.5)
**Ratio (ANG-2/ ANG-1)**	0.00003 (0.000013–0.0021)	0.017 (0.03–0.11)	3.47 (0.15–204)	0.0015 (0.00071–0.014)	0.013 (0.0011–13.0)	0.14 (0.0024–81.5)
**TNF (pg/ml)**	0 (0–0)	0 (0–44.8)	6.51 (0–73.8)	0 (0–31.3)	70.6 (0–658)	76 (0–559)

Values are presented as median (range).

### Receiver operating characteristic (ROC) curves indicate that angiopoietin levels discriminate between uncomplicated and cerebral malaria patients

ROC curves for the biomarkers, examining CM patients as “cases” and uncomplicated malaria patients as “controls”, were plotted and compared to assess the ability of each marker to discriminate between patients with and without cerebral complications (Figure 1&2, Table 3). In the Thai population, ANG-1 and the ANG-2∶ANG-1 ratio have an area under the curve (AUC) of 1 (Figure 1B, Table 3) and differ significantly (p<0.001) from that of a chance result (AUC: 0.5). This finding was validated in the geographically, genetically and demographically distinct Ugandan pediatric population, where ANG-1 (AUC: 0.785, p<0.001) and the ANG-2∶ANG-1 ratio (AUC: 0.779, p<0.001) were still the best of the biomarkers examined (Figure 2B, Table 3; sICAM-1, data not shown). Although ANG-2 did not have such large AUC values, it showed moderate accuracy as a discriminatory marker in both populations examined (Figure 1B - Thai: AUC = 0.835, p<0.001; Figure 2B - Uganda: AUC = 0.688, p<0.001).

**Table 3 pone-0004912-t003:** Area under ROC curve (AUC) for each test comparing UM with CM patients.

Marker	Adult (Thailand)		Pediatric (Uganda)	
	AUC (95% CI)	P	AUC (95% CI)	P
**ANG-1**	1 (1–1)	<0.001	0.785 (0.709–0.861)	<0.001
**ANG-2**	0.835 (0.719–0.951)	<0.001	0.688 (0.595–0.780)	<0.001
**Ratio**	1 (1–1)	<0.001	0.779 (0.702–0.856)	<0.001
**TNF**	0.834 (0.713–0.955)	<0.001	0.557 (0.453–0.661)	0.268

P values are based on the null hypothesis that AUC = 0.5.

Compared to ANG-1 and ANG-2 as biomarkers of CM, previously studied markers of severe and CM such as TNF (Figure 1B, Figure 2B, Table 3) had moderate accuracy as a discriminating test (Figure 1B, AUC: 0.834, p<0.001) in Thai adults; however, TNF was a poor discriminator between CM and uncomplicated malaria in the Ugandan pediatric population (Figure 2B, AUC: 0.557, p = 0.268).

### ANG-1 shows high sensitivity and specificity as a biomarker of cerebral malaria

The diagnostic accuracy (sensitivity, specificity, positive and negative likelihood ratios) for each biomarker, stratified by patient population, are reported in Table 4. Based on ROC curve analysis, ANG-1 best discriminated CM from UM. In the Thai population, ANG-1 at a threshold of 21 ng/mL had a sensitivity and specificity of 100% for distinguishing CM from UM, while for Ugandan children ANG-1 (at a cut off of 15 ng/mL) distinguished CM from UM with sensitivity and specificity of 70% and 75%, respectively. Across both populations, using an ANG-1 threshold of 15 ng/mL, the pooled sensitivity (95% CI) was 0.77 (0.67–0.84), specificity 0.82 (0.72–0.88), likelihood ratio of CM given a positive test (ANG-1 below 15 ng/mL) was 4.1 (2.7–6.5) and the likelihood ratio of CM given a negative test was 0.29 (0.20–0.42).

**Table 4:** Optimal cut-off values (95% CI) for each test and sensitivity (SEN), specificity (SPEC), positive likelihood ratio (LR(+)) and negative likelihood ratio (LR(−)) at the chosen cut-off value comparing uncomplicated malaria with cerebral malaria patients.

Marker	Adult (Thailand)					Pediatric (Uganda)				
	Cut-off	SEN	SPEC	LR(+)	LR(−)	Cut-off	SEN	SPEC	LR(+)	LR(−)
**ANG-1**	21.26 ng/ml	1 (0.87–1)	1 (0.87–1)	∞*	0*	15.05 ng/ml	0.70 (0.58–0.79)	0.75 (0.63–0.83)	2.7 (1.8–4.3)*	0.40 (0.28–0.60)*
**ANG-2**	3.04 ng/ml	0.72 (0.52–0.86)	0.84 (0.65–0.94)	4.5 (1.8–11)*	0.33 (0.17–0.64)*	0.39 ng/ml	0.83 (0.72–0.90)	0.60 (0.48–0.71)	2.1 (1.5–2.8)*	0.29 (0.17–0.51)*
**Ratio**	0.131	1 (0.87–1)	1 (0.87–1)	∞*	0*	0.052	0.73 (0.61–0.82)	0.70 (0.58–0.79)	2.4 (1.6–3.6)*	0.39 (0.26–0.59)*
**TNF**	1.46 pg/ml	0.76 (0.57–0.89)	0.88 (0.70–0.96)	6.3 (2.1–19)*	0.27 (0.13–0.56)*	81.1 pg/ml	0.48 (0.36–0.61)	0.62 (0.49–0.74)	1.3 (0.84–2.0)	0.82 (0.60–1.1)

* significantly different from 1 (p<0.05).

### The association of ANG-1 with CM is independent of parasite burden and other covariates

Although higher parasitemia is generally associated with an increased risk of severe malaria or CM, severe disease can occur in individuals with relatively low peripheral parasitemias. In the Thai population, patients with CM had significantly higher parasitemias than in uncomplicated malaria patients (p<0.001); however, this was not the case in Ugandan children (Table 1). Increased serum cytokine levels may reflect the immune response to increased parasite burdens, rather than being indicative of a clinical syndrome such as CM. In support of this hypothesis, TNF levels were significantly correlated with the parasite burden among Ugandan children with UM (r^2^ = 0.38, p = 0.004) and CM (r^2^ = 0.44, p<0.001). In contrast, angiopoietins did not significantly correlate with parasitemia in an analysis stratified by clinical syndrome and patient population, yet were strongly associated with CM, suggesting that they provide diagnostic information independent of measured parasitemia. Furthermore, ANG-1 (but not TNF) was independently associated with CM in a multivariate logistic regression model, adjusting for the potential confounding effects of multiple covariates (Table 5).

**Table 5:** Results of a multivariate logistic regression model to predict CM (versus UM) in two diverse patient populations.

Predictor	Adjusted OR (95%CI)	p
Group:		
Thailand	1.0*	
Uganda	0.36 (0.029–4.7)	0.44
Age	0.96 (0.86–1.1)	0.53
Parasitemia (parasites/µL)	1.00 (1.00–1.00)	0.20
ANG-1 (ng/mL)	0.899 (0.864–0.934)**	<0.001
ANG-2 (ng/mL)	1.10 (0.944–1.28)	0.22
Ratio (ANG-2/ANG-1)	1.01 (0.932–1.09)	0.82
TNF (pg/mL)	1.00 (0.994–1.003)	0.91

* baseline comparator group.

** Adjusted odds ratio represents the incremental odds of CM for every unit increase (1 ng/mL) in the ANG-1 level.

### ANG-1 levels and the ANG-2∶ANG-1 ratio predict survival in African children with cerebral malaria

**Figure 3:**
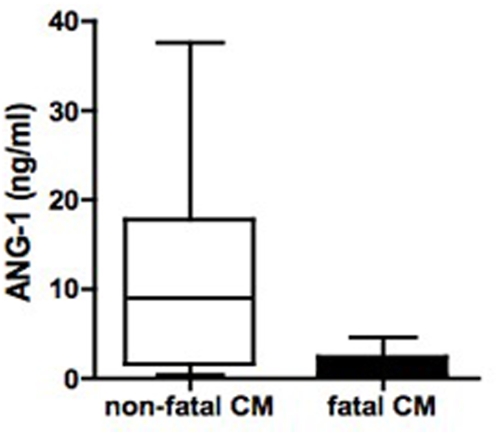
Angiopoietin-1 levels are associated with clinical outcome in pediatric cerebral malaria patients from Uganda. Serum concentrations of angiopoietin-1 (ANG-1) were measured in 69 cerebral malaria (CM) patients at presentation and compared to outcome. Higher ANG-1 levels at presentation were associated with protection from fatal cerebral malaria. ^*^p = 0.027, non-fatal CM versus fatal CM (Wilcoxon rank-sum test).

We examined angiopoietin levels at presentation and subsequent survival in children with CM and observed that ANG-1 levels and the ratio of ANG-2∶ANG-1 were related to mortality. Higher ANG-1 levels at presentation were associated with protection from fatal CM (median (range): non-fatal CM 9.1 (0.39 to 38) versus fatal CM 0.39 (0.39 to 4.6), p = 0.027; Figure 3), whereas ANG-2∶ANG-1 ratios were higher in those who subsequently died of CM (median (range): non-fatal CM 0.13 (0.01 to 82) versus fatal CM 2.6 (1.4 to 13), p = 0.013). No patients died in the Thai cohort.

## Discussion

This study provides evidence implicating dysregulation of angiopoietins in the pathogenesis of CM and suggests that they may be clinically informative biomarkers of this syndrome. Since the manifestations of severe malaria may differ between children and adults and in varying backgrounds, we measured serum ANG-1 and ANG-2 levels in two geographically and genetically diverse patient and parasite populations and demonstrate that these endothelial regulators were accurate discriminators of CM vs. UM in both settings. In both adults from Thailand (Figure 1; Tables 2–4) and children from Uganda (Figure 2; Tables 2–4), low ANG-1 levels or increased ANG-2∶ANG-1 ratios were shown to be informative biomarkers of CM and superior to TNF. Furthermore, ANG-1 levels and the ANG-2∶ANG-1 ratios predicted survival in African children with CM (Figure 3). Our findings are in agreement with a recent study by Yeo *et al.* [33] who reported that ANG-2 levels were higher in Indonesian adults with severe malaria and were better predictors of death than other markers of disease, such as lactate. Our study extends these observations to African children and suggests that the balance between ANG-2 and ANG-1 may be particularly informative with respect to the state of endothelial activation and disease severity.

No laboratory tests are currently available to definitively confirm a diagnosis of CM, and misdiagnosis may result in increased adverse outcomes [34], [35]. The lack of a reference standard for definitive diagnosis of CM is associated with misdiagnosis of CM, particularly in African children where post-mortem studies have shown approximately 20% of “cerebral malaria” cases were due to other causes [1], [35]. The ability to accurately determine the presence of, or risk for progression to CM in falciparum-infected individuals would be of benefit in patient triage, appropriate clinical management and efficient resource allocation. Fundoscopic examination demonstrating malarial retinopathy has been reported to be a useful pre-mortem discriminator of severe malaria and has been proposed as a diagnostic test for CM [35], [36]. However, indirect fundoscopy has inherent limitations, including requirements for pupil dilation, specialized training and equipment. Furthermore, it is unclear whether fundoscopy can be used to predict which children will progress to CM, and retinopathy does not appear to be a consistent feature of CM in adults [36], [37], [38]. Although the current study did not directly address whether ANG-1 and ANG-2 could be used to predict which patients with uncomplicated disease will progress to cerebral malaria, our results clearly demonstrate that ANG-1 and ANG-2 are sensitive and specific indicators of severe disease that effectively differentiate between uncomplicated malaria and CM.

An ideal biomarker for CM might be expected to possess a number of logistical, diagnostic/prognostic and therapeutic attributes, including 1) capacity to be easily measured in a readily available specimen such as serum or whole blood by a standardized assay that requires limited specialized equipment and performed with minimal training, 2) reliable detection, with high sensitivity and specificity of individuals with either established CM or at risk of progression to CM, and 3) detection of determinants likely to be involved in the underlying pathogenesis of the disorder (rather than bystander reactions/epiphenomena), thereby providing a metric of the underlying disease process, as well as representing potential therapeutic targets for intervention.

Despite the growing realization that CM is a complex multisystem disorder, our data suggest that angiopoietins meet several of these criteria and may represent clinically useful biomarkers for this syndrome. Angiopoietins appear to be robust and accessible targets, readily detectable by standard immunoassays in serum or whole blood. ROC curve analysis in both Ugandan pediatric and Thai adult populations indicated that ANG-1 and ANG-2 were highly accurate tests for the detection of CM and its discrimination from uncomplicated disease (Figure 1&2; Table 3), and superior to current markers, such as TNF (Figures 1&2; Table 3) and sICAM-1 (data not shown). In this study, serum TNF levels were positively correlated with parasitemia, whereas angiopoietin levels were not. Although peripheral parasitemia is a limited marker of disease burden in malaria, it does not account for total parasite burden, which includes sequestered parasites [39]. Total parasite biomass can be estimated using plasma HRP-2 [39], and Yeo *et al.* [33] have recently reported that ANG-2 levels were positively correlated with this marker of parasite burden. However, the different relationship between the angiopoietins, TNF and parasitemia in our study suggests that higher levels of TNF may relate to parasitemia whereas the change in ANG-2∶ANG-1 ratio may be related to the overall clinical syndrome of CM. This may be an important distinction given the growing body of evidence supporting an essential role for host-mediated immunopathology and tissue injury in the pathogenesis of CM (reviewed in [24]).

Our observations that ANG-1 and ANG-2 are dysregulated in patients with CM, supports the hypothesis that they may be involved in the pathogenesis of this syndrome. As key regulators of endothelial integrity, there are several mechanisms by which angiopoietins may contribute to the pathophysiology of CM. Although the role of BBB disruption in CM remains controversial [33], [40], [41], CM is characterized by parasite sequestration to CAMs, dysregulated inflammation, and endothelial cell and BBB dysfunction [22], [23], [24], [25], [26]. The endothelium is a large and continuous vascular organ whose state of activation is dependent upon the angiopoietin-Tie2 system [27]. ANG-1 maintains endothelial quiescence and intact tight junctions important for preventing vascular permeability especially across the BBB. ANG-2, stored in endothelial cell granules, may be rapidly released resulting in endothelial activation, augmented inflammation, loosening of endothelial cell junctional complexes, and upregulation of cerebral endothelial adhesion molecules such as ICAM-1 to which parasitised erythrocytes adhere. Increases in BBB permeability have been proposed to be one of the earliest events in the pathogenesis of CM [24]. Therefore dysregulation of angiopoietins, as occurs when ANG-2 levels rise and ANG-1 levels fall, may reflect one of the pivotal or initiating events in the syndrome.

It will be important to dissect the putative mechanisms by which angiopoietins may contribute to malaria pathogenesis in animal models where endothelial and BBB dysfunction and vascular leak are central features of disease [25]. If confirmed by additional studies in humans and clinically relevant animal models, advanced therapies to preserve regulated angiogenic responses, for example by delivering recombinant ANG-1 to restore endothelial cell quiescence, can be examined to determine if they offer clinical benefit as they have in other models of life-threatening infectious disease [42]. It will also be of interest to determine if ANG-2/ANG-1 imbalance will predict outcome in other severe infectious and inflammatory disease states that impact vascular integrity and permeability such as dengue and other viral hemorrhagic fevers, rickettsial infections, toxic shock syndrome and sepsis [43], [44].

One limitation of our study is the relatively small sample sizes, particularly in the Thai population. The sensitivity and specificity of ANG-1 levels and the ANG-2/ANG-1 ratio for the diagnosis of CM was 100% in the Thai population and somewhat lower in the Ugandan pediatric cohort. It will be important to confirm and extend our observations and further assess performance and specificity in larger prospective clinical trials, especially those assessing malarial retinopathy and autopsy studies with histopathologically confirmed cases of CM [35], [36]. Another limitation is that this study focused on the utility of angiopoietin levels in the diagnosis and outcome of CM. Future prospective studies will be required to assess the value of serum angiopoietin levels in predicting progression and outcome of severe or cerebral disease and in distinguishing CM from coma of other causes. With respect to the specificity of angiopoietins for CM versus other life-threatening infections, it is important to note that biomarkers such as angiopoietins are more likely to provide clinically relevant information pertaining to the mechanism and severity of the underlying disease process and the need for critical care triage/referral, and are not expected to be pathogen-specific. Therefore, they will be expected to complement, rather than replace, conventional pathogen diagnosis (for example, microscopy, HRPII or pLDH detection for malaria) and enhance triage and clinical management.

In summary, these data suggest that the dysregulation of angiogenic factors may be involved in the pathogenesis of cerebral malaria and that serum ANG-1 and ANG-2 levels are accurate biomarkers to discriminate CM from uncomplicated disease and predict survival in African children with cerebral involvement.
